# Herbal Weight Loss Pill Overdose: Sibutramine Hidden in Pepper Pill

**DOI:** 10.1155/2015/213874

**Published:** 2015-03-11

**Authors:** Gul Pamukcu Gunaydin, Nurettin Ozgur Dogan, Sevcan Levent, Gulhan Kurtoglu Celik

**Affiliations:** ^1^Ankara Ataturk Training and Research Hospital, Bilkent Yolu 3. Km., Ankara, Turkey; ^2^Department of Emergency Medicine, Faculty of Medicine, Kocaeli University, Umuttepe Kampüsü, Kocaeli, Turkey; ^3^Bilecik State Hospital, Gazipaşa Mahallesi Tevfikbey Caddesi No. 4, Bilecik, Turkey

## Abstract

Supposedly herbal weight loss pills are sold online and are widely used in the world. Some of these products are found to contain sibutramine by FDA and their sale is prohibited. We report a case of a female patient who presented to the emergency department after taking slimming pills. 17-year-old female patient presented to the emergency room with palpitations, dizziness, anxiety, and insomnia. She stated that she had taken 3 pills named La Jiao Shou Shen for slimming purposes during the day. Her vital signs revealed tachycardia. On her physical examination, she was restless, her oropharynx was dry, her pupils were mydriatic, and no other pathological findings were found. Sibutramine intoxication was suspected. She was given 5 mg IV diazepam for restlessness. After supportive therapy and observation in emergency department for 12 hours there were no complications and the patient was discharged home. Some herbal pills that are sold online for weight loss have sibutramine hidden as an active ingredient, and their sale is prohibited for this reason. For people who use herbal weight loss drugs, sibutramine excessive intake should be kept in mind at all times.

## 1. Introduction

A variety of the so-called herbal pills are widely used for slimming purposes in the world. Because these pills are not considered as medicine, there is no standardization for the ingredients of them and they may have hidden ingredients [1]. Serious adverse effects even death have been reported due to herbal pills consumption.

Some slimming pills are thought to be herbal, natural, and healthy, but sibutramine has been detected as an active ingredient in them. In one study ingredients of herbal slimming pills were analyzed and sibutramine was the most often detected synthetic substance [2]. Turkish Ministry of Health has analyzed some supposedly herbal pills that are sold in Turkey (Pepper Time Capsule©, Lida Daidaihua Weight Loss Capsule©, and La Jiao Shou Shen Capsule©) and has found that these pills have contained more sibutramine than the licensed weight loss medicine. The sale of those pills is banned.

Sibutramine is a synthetic noradrenaline, dopamine, and serotonin reuptake inhibitor used for the treatment of obesity. FDA and European Medicines Agency banned it because of its cardiovascular risks [1, 3, 4]. Tachycardia, hypertension, headache, and dizziness have been reported with sibutramine overdose [1, 5].

Although products that contain sibutramine have been banned, they can be easily purchased online. We present a case that took 3 slimming pills and had findings consistent with sibutramine overdose.

## 2. Case Report

17-year-old female patient has presented to emergency room at 2:30 a.m., with palpitation, dizziness, fatigue, and insomnia. In her medical history she did not have any disease, did not have surgery, and was not on any drugs. She stated that she bought pepper capsules named La Jiao Shou Shen© online. Her neighbor who also used it for weight loss recommended the pills to her. The patient took 3 pills in the previous 24 hours. She had the box of the pills ready with her. She has taken the last pill 4 hours before she arrived at emergency room. In her vital signs her blood pressure was 98/62 mmHg, her pulse was 138/min, and her body temperature was 37.5°C. In her physical exam she had tachycardia and anxiety, her oropharyngeal mucosa was dry, and her pupils were mydriatic. There were no other pathological findings. Her EKG showed sinus tachycardia; rate was 142/dk. Complete blood count, liver enzymes, renal function tests, serum electrolytes, thyroid function tests, and creatine kinase were within normal limits. After search online, the name of the product was found to be in the banned weight loss products that were adulterated with sibutramine. Thus sibutramine intoxication was diagnosed. Because she came to the emergency room 4 hours after she took the last pill, we neither placed nasogastric tube nor performed gastric lavage. Supportive therapy was initiated with normal saline 150 mL/hour. 5 mg of diazepam was given IV for anxiety. She was observed in the emergency room for 12 hours. There were no complications, her physical exam and vital signs returned to normal, and she was discharged with instructions to not use the pills again.

## 3. Discussion 

Dietary supplements are not considered medicine and therefore not rigorously tested before they are on market. Some of these pills have synthetic drugs instead of herbal supplements. Sibutramine was detected in 44% of herbal slimming products. Some of these products may have up to 35 mg of sibutramine per pill [6]. In another study 80% percent of the patients who presented with weight loss pill overdose were found to take sibutramine unintentionally and 73% percent of products had sibutramine in them [7]. Consumers are not aware of the threat in many herbal products adulterated with synthetic drugs. Some common drugs hidden in weight loss pills are displayed in Table 1.

The most commonly reported side effects of sibutramine use are headache, dry oropharyngeal mucosa, anorexia, constipation, and insomnia whereas tachycardia, hypertension, headache, and dizziness are reported for its overdose [1, 5]. Serotonin syndrome has also been reported in some cases that were on sibutramine [8]. Sibutramine overdose complications are shown in Table 2.

In two published case reports 2 female patients and a male patient that took the same particular brand of herbal weight loss pill as in our case were presented. Their symptoms were attributed to capsaicin but we think these patients may also have sibutramine intoxication [9, 10].

In this case we could not test blood or urine for sibutramine or could not analyze the ingredients of the capsules unfortunately. Sibutramine intoxication was considered because the symptom onset was rapid and the patient had no disease, was not on any drugs, and her symptoms were consistent with sibutramine intoxication. FDA announced that this particular brand (La Jiao Shou Shen©) of pepper pills contains sibutramine and phenolphthalein [11]. This has also supported our diagnosis.

Some weight loss pills are banned for having sibutramine as an active ingredient but these pills can be easily bought online and sibutramine overdose should always be kept in mind in cases with herbal weight lose pills consumption.

## Figures and Tables

**Table 1:** Common drugs hidden in weight loss pills [1, 7].

Appetite suppressant	Diuretic	Laxative	Other
Ephedrine Fenfluramine Mazindol Sibutramine Pseudoephedrine	Hydrochlorothiazide Spironolactone	Sennosides Phenolphthalein Anthraquinones Bisacodyl	Fluoxetine Thyroid hormones Caffeine

**Table 2:** Sibutramine overdose complications [2].

(i) Cardiovascular: tachycardia, palpitations, chest pain, and hypertension	
(ii) Neurological: headache, anxiety, irritation, dizziness, insomnia, paresthesia, and serotonin syndrome	
(iii) Gastrointestinal: nausea, dry mouth, and constipation	

## References

[1] Ozdemir B., Sahin I., Kapucu H. (2013). How safe is the use of herbal weight-loss products sold over the Internet?. *Human and Experimental Toxicology*.

[2] Kim H. J., Lee J. H., Park H. J., Cho S.-H., Kim W. S. (2014). Monitoring of 29 weight loss compounds in foods and dietary supplements by LC-MS/MS. *Food Additives and Contaminants, Part A: Chemistry, Analysis, Control, Exposure and Risk Assessment*.

[3] FDA Government (2014). *FDA Drug Safety Communication: FDA Recommends against the Continued Use of Meridia (Sibutramine)*.

[4] http://www.ema.europa.eu/docs/en_GB/document_library/Referrals_document/Sibutramine_107/WC500094238.pdf.

[5] FDA.gov Meridia drug information sheet. http://www.fda.gov/downloads/Drugs/DrugSafety/PublicHealthAdvisories/UCM130745.pdf.

[6] Mathon C., Ankli A., Reich E., Bieri S., Christen P. (2014). Screening and determination of sibutramine in adulterated herbal slimming supplements by HPTLC-UV densitometry. *Food Additives and Contaminants Part A: Chemistry, Analysis, Control, Exposure and Risk Assessment*.

[7] Tang M. H., Chen S. P., Ng S. W., Chan A. Y., Mak T. W. (2011). Case series on a diversity of illicit weight-reducing agents: from the well known to the unexpected. *British Journal of Clinical Pharmacology*.

[8] Lam P. K., Leung K. S., Wong T. W., Lee H. H. C., Tang M. H. Y., Mak T. W. L. (2012). Serotonin syndrome following overdose of a non-prescription slimming product containing sibutramine: a case report. *Human and Experimental Toxicology*.

[9] Sogut O., Kaya H., Gokdemir M. T., Nimetoglu M. S., Solduk L. (2010). Cardiotoxicity developed after the use of cayenne pepper pill for slimming: a report of two cases. *Turkish Journal of Emergency Medicine*.

[10] Sogut O., Kaya H., Gokdemir M. T., Sezen Y. (2012). Acute myocardial infarction and coronary vasospasm associated with the ingestion of cayenne pepper pills in a 25-year-old male. *International Journal of Emergency Medicine*.

[11] FDA.gov Public Notification: La Jiao Shou Shen Contains Hidden Drug Ingredient. http://www.fda.gov/Drugs/ResourcesForYou/Consumers/BuyingUsingMedicineSafely/MedicationHealthFraud/ucm400595.htm.

